# Supplementary material for: AmoA-Targeted Polymerase Chain Reaction Primers for the Specific Detection and Quantification of Comammox Nitrospira in the Environment
Source: Front Microbiol. 2017 Aug 4;8:1508. doi: 10.3389/fmicb.2017.01508 (PMC5543084; doi:10.3389/fmicb.2017.01508)
Supplement: Supplementary file 1 [file Table1.PDF]

Table S1. Distribution of comammox *amoA* OTU members in the analyzed environmental samples. For each OTU and sample, the numbers of retrieved *amoA* sequences are indicated.

OTU	Sample ID (see Table 2 in the main text for abbreviations)												
	WB	FH	SP	BH	LS	VM	IS	RAS	VS	VR	KLD	HS	RS
**1**	17	17		2									
**2**								3					
**3**	4												
**4**	1												
**5**	1												
**6**		3		3					1				
**7**	1	1	22										
**8**					7			2					
**9**												5	
**10**												3	
**11**					1								
**12**										1			
**13**						12							
**14**							5						
**15**						1							
**16**									1				
**17**						1							
**18**						5							
**19**									7	2			
**20**													32
**21**													5
**22**												2	
**23**								1					
**24**		3		5									
**25**								1		4			
**26**										4			
**27**										1			
**28**									9	12			
**29**									1				
**30**									5	5			
**31**									2	2			
**32**		19	22	4							6		
**33**									12	13			
**34**									7	4			
**35**								3					
**36**								3					
**37**											1		
**38**	26			2									
**39**		2	1	4									
**40**									8	5			
**41**											4		
**42**									9	5	34		
**43**									5	1			
**44**											10		
**45**	1												
**46**								2					
**47**											1		
**48**											1		
**49**											3		
**50**										1			
