# Supplementary material for: AmoA-Targeted Polymerase Chain Reaction Primers for the Specific Detection and Quantification of Comammox Nitrospira in the Environment
Source: Front Microbiol. 2017 Aug 4;8:1508. doi: 10.3389/fmicb.2017.01508 (PMC5543084; doi:10.3389/fmicb.2017.01508)
Supplement: Supplementary file 2 [file Table2.PDF]

Table S2. Copy numbers of *amoA* genes from comammox *Nitrospira* and canonical ammonia oxidizers in environmental samples as determined by qPCR analysis.

Sample name	Habitat type	Gene amplified	Copies / ng DNA
Wolfenbüttel	Engineered (GWW)	Clade A *amoA*	$1.1 \pm 0.2 \times 10^{4}$
		Clade B *amoA*	$2.5 \pm 0.2 \times 10^{4}$
		AOB *amoA*	$4.0 \pm 2.1 \times 10^{3}$
Friedrichshof	Engineered (DWTP)	Clade A *amoA*	$1.7 \pm 0.3 \times 10^{3}$
		Clade B *amoA*	$3.7 \pm 0.6 \times 10^{3}$
		AOB *amoA*	$4.4 \pm 0.5 \times 10^{4}$
		AOA *amoA*	$1.1 \pm 0.3 \times 10^{5}$
VetMed	Engineered (WWTP)	Clade A *amoA*	$5.1 \pm 1.7 \times 10^{2}$
		AOB *amoA*	$1.7 \pm 0.4 \times 10^{3}$
Vercelli rice soil	Rice paddy soil	Clade A *amoA*	$4.6 \pm 1.0 \times 10^{2}$
		Clade B *amoA*	$4.0 \pm 0.5 \times 10^{2}$
		AOA *amoA*	$2.6 \pm 0.5 \times 10^{3}$
Klausen-Leopoldsdorf	Forest soil	Clade B *amoA*	$3.9 \pm 1.0 \times 10^{2}$
		AOB *amoA*	$2.6 \pm 0.9 \times 10^{3}$
		AOA *amoA*	$2.0 \pm 0.6 \times 10^{2}$
