# Supplementary material for: AmoA-Targeted Polymerase Chain Reaction Primers for the Specific Detection and Quantification of Comammox Nitrospira in the Environment
Source: Front Microbiol. 2017 Aug 4;8:1508. doi: 10.3389/fmicb.2017.01508 (PMC5543084; doi:10.3389/fmicb.2017.01508)
Supplement: Supplementary file 3 [file Image1.PDF]

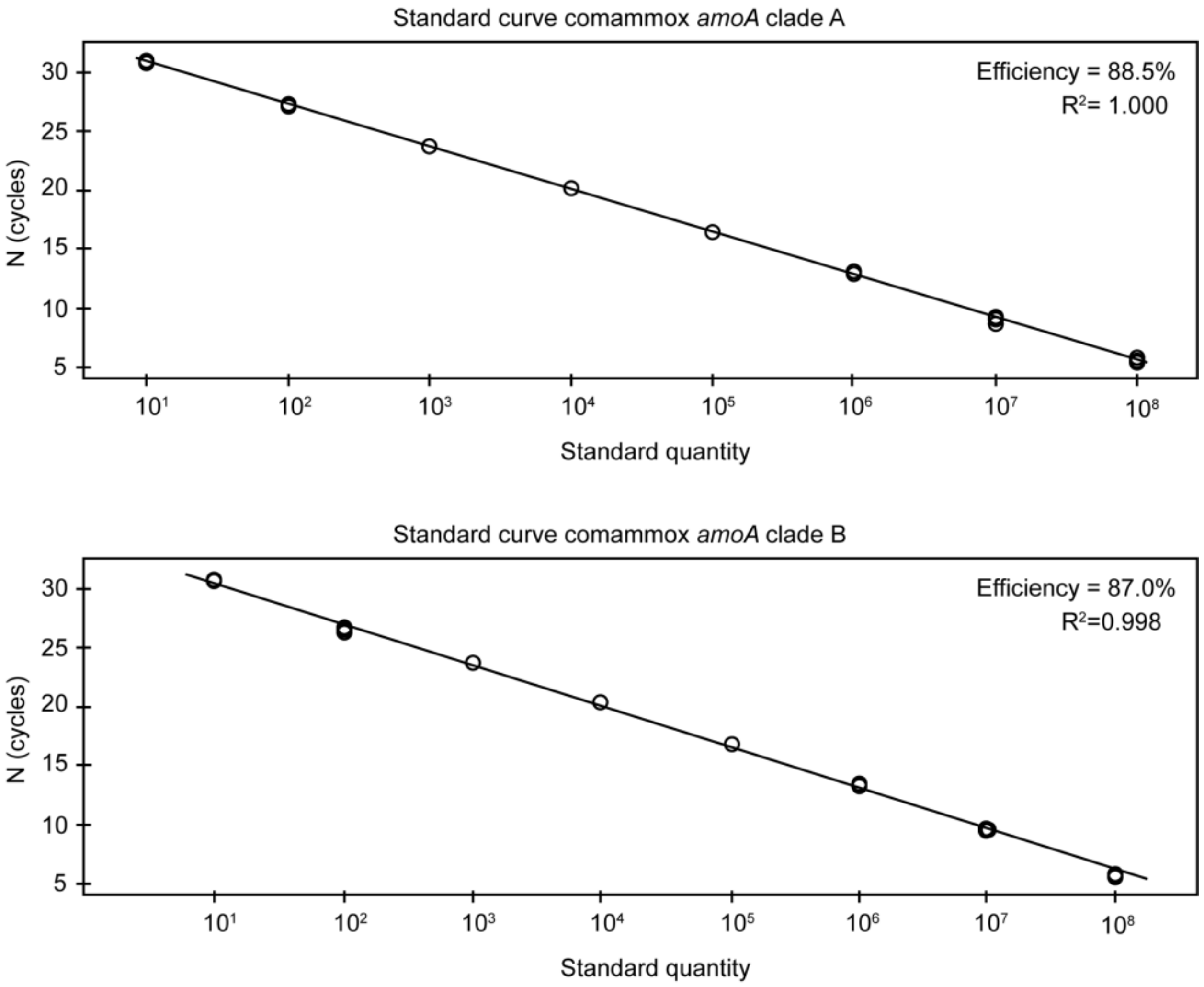

Figure S1. Calibration of comammox *amoA*-targeted qPCR with external standard curves for comammox *amoA* clade A (upper panel) and clade B (lower panel). Please note the logarithmic scaling of the *x* axes. Data points from three replicates are superimposed.
