# Supplementary material for: AmoA-Targeted Polymerase Chain Reaction Primers for the Specific Detection and Quantification of Comammox Nitrospira in the Environment
Source: Front Microbiol. 2017 Aug 4;8:1508. doi: 10.3389/fmicb.2017.01508 (PMC5543084; doi:10.3389/fmicb.2017.01508)
Supplement: Supplementary file 4 [file Image2.PDF]

A

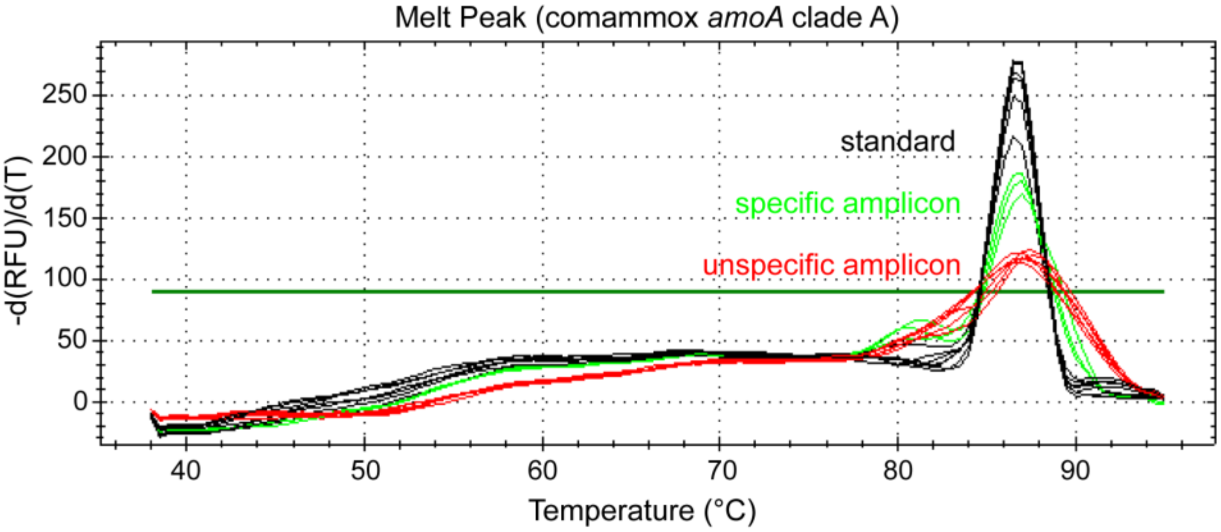

B

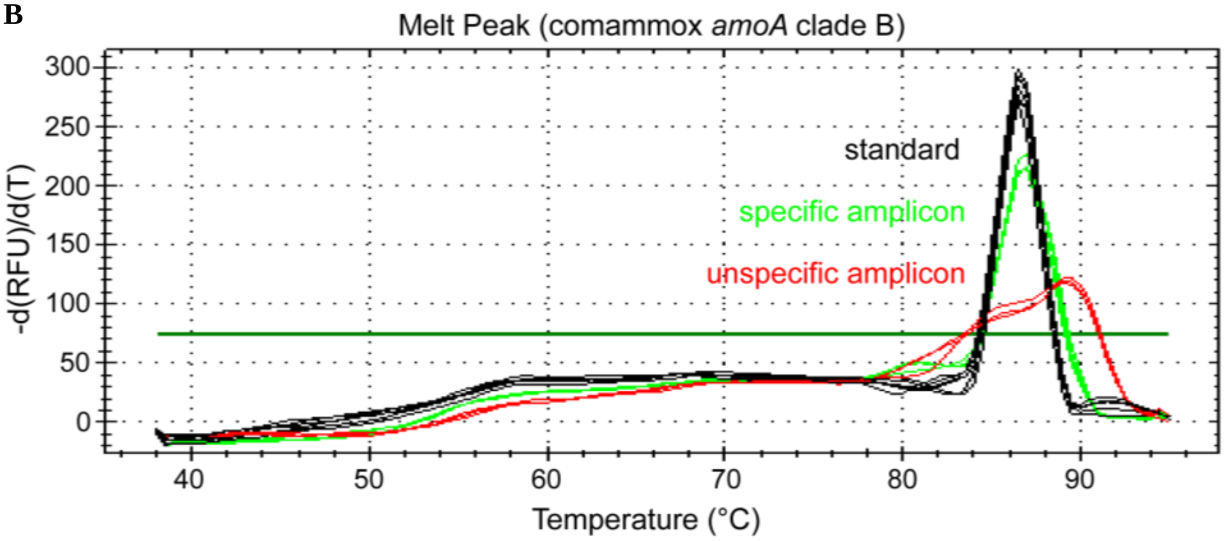

Figure S2. Melting curve analyses of amplicons obtained by comammox *amoA*-targeted qPCR from cloned *amoA* genes (standard) and environmental samples. **A.** Analysis of comammox *amoA* clade A. The specific amplicons were obtained from WWTP VetMed, the unspecific amplicons from the Klausen-Leopoldsdorf forest soil, which did not contain detectable comammox *amoA* from clade A. **B.** Analysis of comammox *amoA* clade B. The specific amplicons were obtained from the Klausen-Leopoldsdorf forest soil, the unspecific amplicons from WWTP VetMed, which did not contain detectable comammox *amoA* from clade B.
